# Postprandial Glycaemic and Insulinaemic Responses after Consumption of Activated Wheat and Triticale Grain Flakes

**DOI:** 10.1155/2019/6594896

**Published:** 2019-01-10

**Authors:** Laila Meija, Guna Havensone, Aivars Lejnieks

**Affiliations:** ^1^Rīga Stradiņš University, Dzirciema iela 16, Riga, LV 1007, Latvia; ^2^Pauls Stradins Clinical University Hospital, Pilsonu Street 13, Riga, LV 1002, Latvia; ^3^Riga Eastern Clinical University Hospital, Hipokrata Street, Riga, LV 1038, Latvia

## Abstract

Increasing evidence shows that whole grain consumption is protective against metabolic disorders. Protective bioactive substances of whole grains include fibre and antioxidants. Activation of grains can increase the amount of phenolic compounds and their bioavailability, but there is little evidence about their effect on glycaemic and insulinemic responses. Therefore, the aim of this study was to investigate glycaemic and insulinemic responses after consumption of flakes made from activated wheat and activated triticale grains. Eighteen healthy subjects (7 men and 11 women) were given activated wheat or activated triticale flakes. As a reference, a standard glucose solution was used. Plasma glucose and serum insulin were measured during 120 minutes. Both, activated wheat and activated triticale flakes, show improved glycaemic profile, inducing a lower and more stable glycaemic response. However, statistically significant differences in insulin response were found only in the group who have taken activated triticale flakes and not in the group who have taken activated wheat flakes. Activated triticale flakes induced lower insulin response in all postprandial phases and a more stable concentration of insulin. Thus, activated triticale flakes could be beneficial for the prevention and treatment of metabolic disorders.

## 1. Introduction

Whole grain consumption is associated with a decreased risk of all-cause mortality [1, 2]. Moreover, increasing evidence shows an inverse association of whole grain consumption and adiposity [3], coronary heart disease [4], metabolic syndrome, diabetes mellitus [5], and certain types of cancer [6]. The protective mechanism of whole grains can be possibly attributed to many components such as soluble and insoluble fibres [7], vitamins, minerals, and phytochemicals, including lignans, alkylresorcinols, and polyphenols [8]. High fibre content causes a lower energy density of whole grains, which results in increased satiety [9]. Reduced energy intake is associated with lower body mass index [3]. In addition, dietary fibres in whole grains improve glycaemic control by reducing the glycaemic index of the product, providing a slower postprandial glucose increase and reducing postprandial insulin responses [10]. All these factors are essential in the prevention and treatment of metabolic syndrome and type 2 diabetes [11].

One approach to increase bioavailability of grains is activation or germination of grains, which is a method that can be used to improve the nutritional, functional, and sensory properties of cereal grains [12, 13]. The conditions needed for grain activation are optimal humidity, availability of oxygen, and adequate temperature. Biological activation of grains is a process of partial hydrolysis of starch, proteins, cellulose, and hemi-cellulose, resulting in an increased amount of digested compounds and decreasing the activity of antinutritional factors [12]. Therefore, the content of various compounds is affected. Some compounds, like glucans, are degraded, but others, such as antioxidants, total phenolic content, and radical scavenging activity are increased and more available [13, 14]. Activated hydrolytic enzymes increase the amount of digestible vitamins, oligosaccharides, minerals, and amino acids by degrading starch, nonstarch polysaccharides, and proteins, which leads to generation of biofunctional substances [12, 15]. It is known that the type of carbohydrate in diet plays an essential role to glycaemic and insulin responses. However, there are limited data about changes in the contents of individual carbohydrates during germination. It is known that during grain activation within the process of saccharification, complex carbohydrates are degraded into simple sugars. The amount of gluten decreases, but dietary fibre and sugars increase [12].

Wheat is one of the most commonly used grain, with proved benefits if it is used as a whole grain. Triticale (X *Triticosecale* Whittmack) is a hybrid of wheat (*Triticum aestivum* L) and rye (*Secale cereale* L), which combines qualities from both species. Several studies investigated potential biofunctional substances of triticale such as special proteins, starches, *β*-glucan, pentosans, soluble and insoluble fibre, and tocols [16]. Moreover, the amount of dietary fibre and lignans in triticale may be high enough for health benefit [17]. But there are negligible data about the effects of activated grain and grain flakes on postprandial glycaemia and insulinemia. In general, there is increasing interest about activation of grains and possible use as protective functional food [18] and very limited knowledge about flakes made from activated grains and their impact on the metabolic syndrome.

The aim of this study was to investigate glycaemic and insulinemic responses after the consumption of flakes made from activated wheat and activated triticale grains.

## 2. Materials and Methods

### 2.1. Carbohydrate Analyses of Grain Flakes

#### 2.1.1. Plant Material

The grains of conventionally grown wheat (cv ‘Elvis') cultivated at the Institute of Agricultural Resources and Economics (Latvia), and triticale (cv ‘Tulus') at Norwegian Institute of Bioeconomy Research (NIBIO) (Norway). Activated flakes were prepared in the laboratory of the Latvia University of Agriculture, the Faculty of Food Technology.

#### 2.1.2. Grain Activation

For grain activation, the whole grains were cleaned, washed, and soaked in water at the ratio of 1 : 2 (grains to water) for 24 ± 1 hour at 22 ± 2°C. Then, grains were placed for activation in the climatic chamber ICH110 (Memmert, Germany) at the controlled temperature (35 ± 1°C), with relative humidity of 95 ± 2% in the dark. The duration of grain activation was 24 hours [14]. Thereafter, the activated grains were processed into cereal flakes.

#### 2.1.3. Cereal Flakes Preparation

Activated grains were flaked using a manual flaker (Eschenfelder, Germany). The thickness of the obtained flakes was 1.2 ± 0.3 mm. Then, flaked cereals were dried using a microwave-vacuum system “Musson-1” (OOO Ingredient, Russia). Five kilograms of flaked cereals were processed during 56 ± 2 min at 40 ± 5°C.

#### 2.1.4. Chemical Analyses

Chemical analyses of cereal flakes were carried out in the lab of Eurofins Polska. Prior to all analyses, the samples were milled and homogenized. The analysis of dietary fibre and resistant starch content in foods was performed after hydrolysis of carbohydrates in each sample, which was accompanied by proteolysis to remove the protein surrounding starch and make the starch susceptible to amylase hydrolysis. The cereal samples were analysed by the Prosky method [19, 20] for fibre content according to standards of AOAC 991.43 [20] based on an enzymatic-gravimetric procedure Megazyme K-TDFR (Megazyme International, Ireland). Starch content of cereal flakes was determined by Megazyme with a total starch kit K-TSTA (Megazyme International, Ireland) by a spectrophotometric procedure. The contents of total sugars (glucose, fructose, saccharose, lactose, and maltose) were determined according to the method by AOAC 923.09, by a titrimetric procedure [21]. The resistant starch was detected according to the AOAC method 2002.02 by enzymatic hydrolysis followed by spectrophotometric detection [22].

### 2.2. Experimental Part

#### 2.2.1. Subjects

The study started with 21 participants, whereas 3 participants dropped out due to personal reasons. The study was completed with 7 men and 11 women. Participants were randomly assigned to one of the two groups (each with 9 subjects). Both groups were healthy and nonsmoking volunteers aged 22.4 ± 3.7 years, with a body mass index (BMI) of 22.3 ± 2.9 kg/m^2^. The study subjects were recruited among students of the universities of the city of Riga, residing in the city or in the immediately surrounding region. All of them had normal fasting blood glucose concentrations. Subjects were eating habitual food and did not use any medicine or food supplements or functional food that could have influenced glycaemia. The study period was between September and November 2016. All procedures were approved by the Research Ethics Committee of Pauls Stradins University, and all the subjects signed the informed consent. Data on age, body weight, and height were self-reported.

#### 2.2.2. Experimental Design

**Figure 1:**
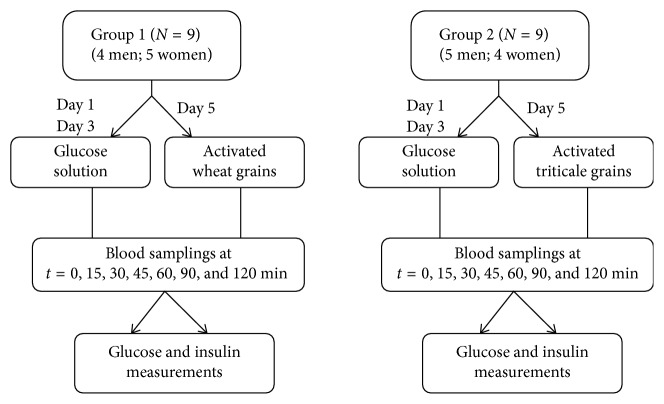
The study design.

The study design is shown in Figure 1. The testing process was carried out during 1 week. The participants arrived at the laboratory at 8 : 00 a.m. after 10–12 hours of overnight fasting. They were asked to avoid foods that are rich in dietary fibres (e.g., legumes) the day before the test day. They were also asked to avoid alcohol and excessive physical exercise in the evening before the experiment. The first, control blood sample (at *t* = 0) was taken in the fasting status. The concentration of glucose was determined in the capillary blood and the concentration of insulin in the peripheral venous blood. After the consumption of the meal, blood samples were taken at six times at 15, 30, 45, 60, 90, and 120 minutes. The participants were examined 3 times. The first two times the reference meal was used. At the 3rd visit, activated wheat or triticale flake meal was given. The participants were instructed to finish their meal within 10–15 min.

#### 2.2.3. Test Meals

The test meals consisted of test food (activated wheat flakes or activated triticale flakes) or of reference (glucose solution). All meals provided 50 g of available carbohydrates. The reference meal contained 50 g of glucose that was dissolved in 250 ml water. The mass of activated wheat flakes was 107 g and the mass of activated triticale flakes was 99 g. The available amount of carbohydrates was calculated as the sum of free sugars and enzymatically available starch according to the methodology by Broun et al. [23]. In addition, 250 ml of water was included in each cereal flake meal. The ways of production of activated grain flakes were described above.

#### 2.2.4. Laboratory Methods

Capillary blood was taken with Sarstedt glucose microvette that contains 200 *μ*l sodium fluoride. Glucose determination was done by the hexokinase method with an analyser Architect c8000 (ABBOTT) and an intra assay coefficient of variation <3%.

Blood samples (4 ml) were collected by venepuncture using the Greiner bio-one vacuette that contains an anticoagulant, spray-dried lithium heparin. The collected samples were ultracentrifuged immediately. Centrifuged serum samples were assayed for 1–2 hours. Serum insulin was measured by a chemiluminescent immunometric assay with the analyser Immulite 2000 Systems (The Quality System of Siemens Healthcare Diagnostic Products Ltd.). The CVs were 3.0% and 3.5% within- and between-assay, respectively. The samples collected at each time point were analysed within the same run.

### 2.3. Data Analysis

The blood glucose and insulin statistics were analysed with 9 participants in both groups. The inclusion of ten subjects provides a reasonable degree of power and precision [23]. Results were expressed as means ± SD. Cumulative changes in postprandial plasma glucose and serum insulin responses for each meal were quantified as the incremental area under the curve (iAUC), which was calculated by using the trapezoidal rule with fasting concentrations as the baseline, and truncated at zero [23]. Incremental peak concentrations were calculated for glucose and insulin as individual maximum postprandial increase from the baseline.

The difference of significance in glucose and insulin responses (*p* < 0.05) between the products at different time points were evaluated by the Wilcoxon test for paired observations by using the SPSS 22.0.

## 3. Results and Discussion

Table 1 shows the amount of starch, resistant starch, and soluble and insoluble fibres in the flakes of activated wheat and triticale.

Activated triticale flakes contain a larger extent of insoluble fibre. There are few data available about the content of different carbohydrates in different grain flakes. There is even less information available about the content of different carbohydrates in germinated grains or grain products, such as flakes. Average ordinary wheats contain 11.5–18.3% of the total dietary fibre in dry matter [24]. Triticale dietary fibre profile is normally with a total dietary fibre content of 13−16% [25]. More than 80% of dietary fibres in triticale and wheat grain are insoluble fibre [16, 25, 26]. The content of insoluble and soluble nonstarch polysaccharides in triticale grain varied from 7.7 to 9.1% and 1.5–2.8%, respectively [16]. Our results from analysed activated flakes show a similar amount of total fibre and soluble fibre in flakes; however, the content of insoluble fibre was lower in activated wheat flakes (6.8 % wet weight).

It has been shown that wheat contains less resistant starch (3.9%) than triticale (5.3%) [27]. Our data showed a much lower content of resistant starch (1.1% in activated wheat flakes and 0.2% in activated triticale flakes). Usually, the content of resistant starch increases and soluble fibre components decreases during processing [28]. Flakes used in the current study contain a low amount of resistant starch. This could be explained by flake processing procedures used in our study (cereal activation for only 24 h and flakes preparation in low temperature), which is probably too short for phytochemical changes in grains and presumably resulted in low levels of amylose retrogradation.

### 3.1. Plasma Glucose Responses

**Figure 2:**
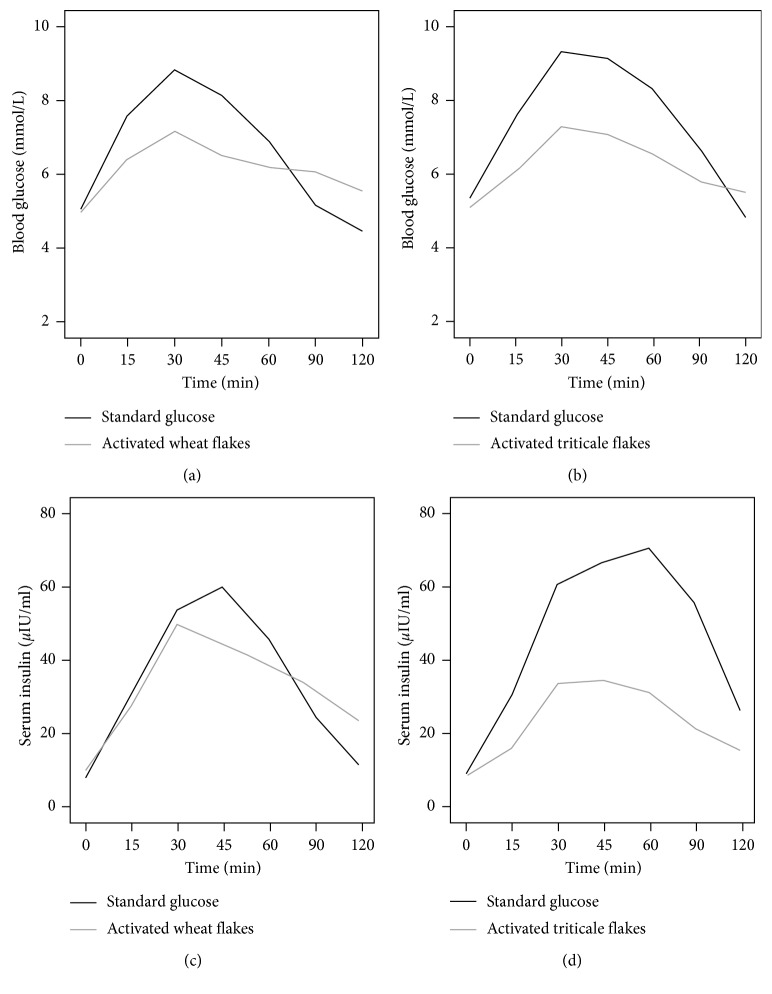
Incremental plasma glucose and serum insulin response (*n*=9). (a) Incremental change in blood glucose in activated wheat flakes. (b) Incremental change in blood glucose in activated triticale flakes. (c) Incremental change in serum insulin in activated wheat flakes. (d) Incremental change in serum insulin in activated triticale flakes. Values are means. In (a), (b), and (d), significant difference between standard glucose and activated flakes was found (*p* < 0.05).

Tables 2 and 3 and Figure 2 show that both activated wheat (Figure 2(a)) and activated triticale flakes (Figure 2(b)) induced more gradual initial rise and significantly lower blood glucose response than reference glucose solution. Both test meals produced lower blood glucose peak values than glucose solution. For activated wheat grain flakes and standard glucose solution, the maximum in the glucose response was in 30 min (respectively, 7.2 ± 1.0 and 8.8 ± 0.7 mmol/L), with a statistically significant difference (*p*=0.007). For activated triticale grain flakes, the maximum was also at 30 min (respectively, 7.1 ± 0.7 and 9.3 ± 0.7 mmol/L) and with a statistically significant difference (*p*=0.005) as well. Activated wheat and triticale flakes showed more prolonged and lower postprandial blood glucose response than the corresponding glucose solution. These findings are consistent with those of previous studies showing that whole grains are characterized by a more beneficial blood glucose profile [5].

Significant differences in blood glucose for each test food were quantified as the incremental area under the 120 min response curve. iAUC were observed at all time periods (Tables 2 and 3). Activated grain flakes elicited lower blood glucose response than the reference glucose solution until the late phase (90 min for activated wheat flakes and 120 min for activated triticale grains). In the late phase, 90–120 min glucose solution showed a sharp postprandial decline and a drop below the fasting concentration at *t* = 0. In contrast, intake of activated cereal flakes showed significantly slower postprandial decrease and more stable concentration of blood glucose levels.

Both flakes showed an improved glycaemic profile and induced a more gradual initial rise and a lower glycaemic response, as well as longer and stable postprandial blood glucose response than the reference glucose solution. These findings are important because the avoidance of frequent hyperglycaemia and consecutive hypoglycaemia will result in a lower and prolonged blood glucose response and reduce oxidative stress and subclinical inflammation, which are important mechanisms in the pathophysiology of the metabolic disorders [29].

### 3.2. Serum Insulin Responses

The insulin response revealed similar tendencies as the blood glucose response. Figures 2(c) and 2(d) show that both activated cereal flakes showed more gradual initial rise of insulin, a lower and more stable insulin response, and produced lower insulin peak values than glucose solution as well. The lower acute insulin demand was associated with the higher fullness and satisfaction. In support of this, meal studies with rye products in healthy adults demonstrated that a low insulin response was associated with less accentuated late postprandial hypoglycaemia and a decreased sense of hunger [30]. Insulin maximal level for activated wheat grain flakes was at 30 min, but for the standard glucose solution, it was at 45 min (respectively, 49.8 ± 43.4 and 69.2 ± 41.3 microIU/mL). Activated triticale flakes induced a lower peak insulin value than the activated wheat flakes. The maximal concentration of insulin for activated triticale grain flakes was found at 45 min and for the standard glucose solution at 60 min (respectively, 34.8 ± 16.0 microIU/mL and 72.92 ± 43.0 microIU/mL).

However, no significant differences in iAUC at all time periods were found at any time point between activated wheat flakes and the glucose solution. In turn, significant differences were found in insulin iAUC at all time periods between activated triticale flakes and the glucose solution (Tables 2 and 3).

More important, significant differences in insulin response of a reference glucose solution was found in activated triticale flakes but not in activated wheat flakes. In addition, activated triticale grains produced a lower insulin peak value. A lower incremental insulin peak of triticale flakes could be anticipated to possess appetite regulating properties and may therefore contribute to a lowered risk of obesity, type 2 diabetes, and cardiovascular disease [30].

These findings are consistent with those of previous studies, but despite the described potential health benefits of activated grains, there are negligible data about activated grain flakes effects on postprandial glycaemia and insulinaemia. No data are available on the glycaemic and insulinaemic responses of triticale flakes. It is assumed that different food factors are responsible for the differences in glycaemic and insulin response. One of the most important factors is the carbohydrate content. In the current study, the content of total sugars, starch, total fibre, and soluble fibre was comparable in both the flakes. The only essential difference was the insoluble fibre concentration that was higher in activated triticale flakes than in activated wheat flakes. It is possible that insoluble fibre plays a role in modulating the insulinaemic response. It is proposed that nonstarch polysaccharides, storage lipids, and storage proteins in the grain endosperm matrix may contribute to lowering the glycaemic and insulinaemic responses; the mode of action has not yet been clearly explained [26, 31]. Actually, meal carbohydrates influence directly the early postprandial blood glucose response, whereas the late blood glucose levels are linked mainly to the effect of insulin and glucagon, as well as a free fatty acids response [5].

## 4. Conclusions

Activated triticale flakes induced a lower glucose and insulin response in all postprandial phases and a more stable concentration of glucose and insulin level than the reference glucose solution, whereas activated wheat flakes induced only an improved glycaemic, but no insulin profile. The beneficial effects could be associated with multiple components, such as insoluble and soluble fibre, antioxidants, and anti-inflammatory substances. The mode of action is supposed to be synergetic, including a slower digestion rate as well as the effects on the gut fermentation processes, which are beneficial for glucose homeostasis and insulin sensitivity. Thus, there are indications that triticale flakes could be an advantageous part of diet for the prevention and treatment of metabolic disorders.

## Figures and Tables

**Table 1:** Mean carbohydrate content (expressed in % ± SD) in activated wheat and triticale grain flakes.

Test variables	Activated wheat flakes	Activated triticale flakes
	Wet weight	Wet weight
Total sugars	3.7 ± 0.4	3.8 ± 0.5
Starch content	49.2 ± NA	51.6 ± NA
Resistant starch	1.1 ± NA	0.2 ± NA
Fibre content	10.1 ± 1.7	10.3 ± 1.8
Soluble dietary fibre	2.8 ± 0.2	2.9 ± 0.2
Insoluble dietary fibre	6.8 ± 0.2	10.0 ± 0.2

NA, not available.

**Table 2:** Plasma glucose response, expressed as iAUC (in mmol∗min/L), and serum insulin response, expressed as iAUC (in microIU∗min/mL) after a meal of activated wheat flakes or a glucose standard.

Test variables	Activated wheat flakes	Glucose standard	Significance *p*
*Plasma glucose response*			
Fasting concentration (mmol/L)	5.0 ± 0.3	5.1 ± 0.3	
iAUC 0–15 min	10.7 ± 3.3	19.0 ± 3.5	0.001
iAUC 0–30 min	24.2 ± 6.2	42.6 ± 6.3	<0.001
iAUC 0–45 min	38.2 ± 9.8	68.3 ± 9.6	<0.001
iAUC 0–60 min	48.6 ± 12.5	86.7 ± 12.9	0.001
iAUC 0–90 min	65.8 ± 15.6	101.7 ± 21.1	0.002
iAUC 0–120 min	78.1 ± 16.0	104.2 ± 25.6	0.012
*Serum insulin response*			
Fasting concentration (microIU/ml)	9.7 ± 9.6	9.3 ± 7.2	
iAUC 0–15 min	132.8 ± 95.3	218.4 ± 121.6	0.122
iAUC 0–30 min	349.5 ± 261.9	534.5 ± 312.7	0.171
iAUC 0–45 min	629.2 ± 464.6	966.0 ± 570.4	0.171
iAUC 0–60 min	868.7 ± 597.2	1360.7 ± 758.4	0.102
iAUC 0–90 min	1255.6 ± 779.5	1862.0 ± 952.3	0.102
iAUC 0–120 min	1525.7 ± 897.3	2056.6 ± 1046.5	0.171

iAUC: incremental area under curve; values are means ± SD; *n* = 9; the *p* value indicate the statistically significant difference between activated wheat flakes and glucose standard solution.

**Table 3:** Plasma glucose response, expressed as iAUC (in mmol∗min/L), and serum insulin response, expressed as iAUC (in microIU∗min/mL) after a meal of activated triticale flakes or a glucose standard.

Test variables	Activated triticale flakes	Glucose standard	Significance *p*
*Plasma glucose response*			
Fasting concentration (mmol/L)	5.1 ± 0.4	5.3 ± 0.2	
iAUC 0–15 min	7.2 ± 3.1	17.6 ± 6.4	<0.001
iAUC 0–30 min	18.8 ± 6.4	40.9 ± 11.9	<0.001
iAUC 0–45 min	34.1 ± 10.6	68.5 ± 18.1	0.002
iAUC 0–60 min	46.9 ± 15.7	91.7 ± 27.9	0.004
iAUC 0–90 min	63.3 ± 25.8	121.5 ± 44.0	0.012
iAUC 0–120 min	71.8 ± 32.8	128.9 ± 49.3	0.011
*Serum insulin response*			
Fasting concentration (microIU/ml)	8.5 ± 4.2	10.3 ± 4.4	
iAUC 0–15 min	58.7 ± 34.1	174.8 ± 116.2	0.009
iAUC 0–30 min	182.0 ± 86.9	466.9 ± 265.3	0.012
iAUC 0–45 min	377.9 ± 178.9	878.3 ± 413.6	0.004
iAUC 0–60 min	560.8 ± 266.4	1306.1 ± 599.5	0.001
iAUC 0–90 min	825.3 ± 383.5	2090.3 ± 1105.5	0.004
iAUC 0–120 min	980.6 ± 459.7	2563.4 ± 1438.7	0.012

iAUC = incremental area under curve; values are means ± SD; *n*=9; the *p* value indicate the statistically significant difference between activated triticale flakes and glucose standard solution.

## Data Availability

Access to these data will be considered by the author upon request.
